# Short-Term Effects of Workstyle Reform for Japanese Doctors on Their Surgical Productivity

**DOI:** 10.7759/cureus.102095

**Published:** 2026-01-22

**Authors:** Yoshinori Nakata, Yuichi Watanabe, Akihiko Ozaki

**Affiliations:** 1 Department of Anesthesiology, Teikyo University Hospital, Tokyo, JPN; 2 Institute of Developing Economies, Japan External Trade Organization, Tokyo, JPN; 3 Department of Surgery, Jyoban Hospital, Fukushima, JPN

**Keywords:** japan, malmquist index, productivity, surgery, workstyle reform

## Abstract

Background: The purpose of this study was to evaluate whether the workstyle reform, aimed at improving physician health and safety, achieved a secondary goal of enhancing surgical productivity by applying the Malmquist Index (MI) model. We hypothesized that the reform would lead to a significant improvement in surgical total factor productivity in the short term.

Methods: We conducted a retrospective observational study at a university hospital, analyzing 1,557 surgical procedures performed by 72 surgeons from April 1 through May 31 in 2023 and 2024. A non-radial, non-oriented MI model was applied under variable returns-to-scale assumptions. Each decision-making unit (DMU) was defined as the most senior-ranking surgeon for a given procedure. Inputs included (1) the number of assisting physicians and (2) the duration of surgery from skin incision to closure. The output was defined as the surgical fee assigned to each procedure. Surgical procedures in 2023 were defined as before workstyle reform, and those in 2024 were after workstyle reform. Inputs and outputs were aggregated for each DMU per year. The primary outcome was the Malmquist Index, with secondary outcomes being the catch-up (CU) and frontier-shift (FS) effects.

Results: There was no statistically significant change in overall surgical productivity between 2023 and 2024 (p = 0.39). Neither the catch-up effect nor the frontier-shift effect showed a significant deviation from zero.

Conclusions: The workstyle reform in 2024 in Japan did not lead to a measurable change in fee-based surgical productivity in its first two months at the tertiary hospital. Neither frontier-shift nor catch-up effect was significant.

## Introduction

Background/rationale

Workstyle reform for Japanese doctors is a priority of Japanese labor policy. It capped the legal overtime of doctors, although this level still poses risks to doctors’ health [1]. A law for workstyle reform for general workers originally became effective on April 1, 2019, and new regulations for workstyle reform based on the law were applied to all fully licensed physicians in Japan on April 1, 2024 [2]. This regulation will be in place through 2035. Japanese doctors have worked very long hours. The virtue of working hard has sustained Japanese healthcare, which is threatened by a shrinking workforce and an aging population. The long work hours of doctors had an international impact because they led to mass demonstrations and general strikes of young doctors and partially paralyzed the healthcare systems in the UK, South Korea, and other countries [3]. Young Japanese doctors tend to avoid selecting surgical specialties; the number of surgeons and obstetricians has not recently increased [1]. The long work hours of doctors adversely affect their health and increase the risk of medical errors [4,5]. This workstyle reform is expected to increase their productivity and efficiency to sustain the present workload and quality of healthcare by reducing the risk of medical errors and by improving doctors’ health [4,5]. Although the reform’s primary goal was health and safety, productivity effects are secondary and uncertain. There is no study on the effects of the workstyle reform for doctors on their productivity.

Objective, quantitative approaches to measuring efficiency and productivity have progressed substantially in the disciplines of economics, business administration, and engineering since 2000 [6,7]. Among these approaches, data envelopment analysis (DEA) is widely used for evaluating efficiency, whereas the Malmquist Index (MI) represents a dynamic extension of DEA. The MI model measures productivity variation between two time points and is expressed as the product of the catch-up (CU) effect and the frontier-shift (FS) effect, enabling identification of the underlying sources of productivity change [8].

Objectives

The purpose of this study was to evaluate whether the workstyle reform, aimed at improving physician health and safety, achieved a secondary goal of enhancing surgical productivity in the short term by applying the MI model. We hypothesized that the workstyle reform targeting physicians would lead to a significant improvement in surgical total factor productivity by decreasing the likelihood of medical errors and enhancing physicians’ overall health status.

## Materials and methods

Study design

This study employed a retrospective observational design. Ethical approval for this series of investigations on surgical efficiency and productivity was obtained from the Institutional Review Board of Teikyo University (approval number: 12-030-4). Due to the retrospective design, the requirement for informed consent was waived. The study was reported in accordance with relevant EQUATOR Network guidelines, particularly the Strengthening the Reporting of Observational Studies in Epidemiology (STROBE) statement [9].

Setting

The research was carried out at Teikyo University Hospital, located in metropolitan Tokyo, Japan. The hospital provides medical services to approximately 1,000,000 residents in the surrounding metropolitan area. It has a capacity of 1,152 beds and performs roughly 9,000 surgical procedures annually across 13 surgical specialties. Teikyo University Hospital is categorized as “A Standard” of this workstyle reform, which limits the annual overtime work hours of each doctor to less than 960 hours after the reform. Other community and non-teaching hospitals may allow up to 1,860 hours of annual overtime work hours per physician. Teikyo University Hospital is under the strictest restriction on the overtime of the doctors, which would reflect the largest effects of the workstyle reform [10].

Participants

We analyzed all surgical procedures conducted in the main operating rooms of Teikyo University Hospital between April 1 and May 31 in both 2023 and 2024. Data were extracted from the hospital’s electronic medical record system. The study period was selected based on two important events: the implementation of the workstyle reform on April 1, 2024, and the revision of the national fee schedule on June 1, 2024. Consequently, the interval from April 1 to May 31 in 2024 allowed assessment of the reform’s effects without confounding from fee schedule changes [11,12]. Surgeries conducted in 2023 served as a control group and were categorized as before workstyle reform, while those in 2024 were classified as after workstyle reform.

The study applied the following exclusion criteria. (1) Procedures performed under local anesthesia by surgeons were excluded due to significant differences in resource use, which hinder meaningful comparison with major operations. Additionally, surgeries from the oral, ophthalmic, and dermatological fields were excluded because these are often minor and typically performed without anesthesiologist involvement. Even when general anesthesia was used in these procedures, they did not reflect the standard activities of those surgeons. (2) Cases where patients died within one month post-surgery were excluded to ensure outcome quality consistency. (3) Surgical procedures not reimbursed under the national surgical payment system in either year were excluded. (4) Procedures with incomplete data were also excluded [13].

Variables

The Malmquist Index (MI) was used to assess dynamic productivity changes in decision-making units (DMUs) across the two study periods; this index is a form of comparative static analysis [6,7]. In this context, a DMU refers to an entity responsible for converting inputs into outputs. MI is derived from data envelopment analysis (DEA), which evaluates DMUs relative to a static efficiency frontier within a given period. MI allows for comparison across periods by breaking productivity change into two components: the catch-up (CU) effect, which reflects internal efficiency gains, and the frontier-shift (FS) effect, which represents technological progress or regression [6]. The productivity change of a DMU between Periods 1 and 2 is mathematically represented as follows [8]:

$CU =(Efficiency of the DMU in Period 2 relative to the Period 2 frontier)/(Efficiency of the DMU in Period 1 relative to the Period 1 frontier)$ $FS =((Efficiency of the DMU in Period 1 relative to the Period 1 frontier)/(Efficiency of the DMU in Period 1 relative to the Period 2 frontier)&times;(Efficiency of the DMU in Period 2 relative to the Period 1 frontier)/(Efficiency of the DMU in Period 2 relative to the Period 2 frontier))^(1/2)$ $MI=CU&times;FS$

In essence, MI captures changes in total factor productivity, reflecting both shifts in operational efficiency (CU effects) and the evolution of the technological frontier (FS effects) [8].

Data sources/measurement

We employed a non-radial, non-oriented Malmquist model assuming variable returns to scale [8,14]. Each DMU was defined as the most senior surgeon participating in a given procedure. This was determined by the academic rank; the surgeon with the highest academic rank was defined as the most senior surgeon. Inputs included the number of assisting physicians (assistants) and the operative time from skin incision to closure (surgical duration). The assisting physicians included other surgeons and surgical trainees (residents and fellows) who have medical licenses. They did not include surgical assistants with other professions (nurses and others). The output was the assigned surgical fee. Surgical procedures were identified by “K codes” (K000-K915) from the Japanese fee schedule, which standardizes fees irrespective of surgeon experience, assistant numbers, or surgical duration [11,12]. Other charges, such as those for anesthesia, transfusion, medications, or special materials, were excluded. All monetary values were converted from Japanese yen to US dollars using a fixed exchange rate of $1 = ¥150 for clarity in international interpretation.

Bias

By including all eligible surgeries within identical calendar periods (April 1 to May 31) across both years, we aimed to eliminate potential seasonal variations, thereby minimizing systematic bias.

Study size

Judging from the annual surgical volume of Teikyo University Hospital, our sample size for the study period of April to May in two years was expected to be large enough to minimize the risk of type II errors. If we assume that 15% improvement in productivity is clinically and managerially relevant, the possibility of type II error becomes lower than 20% with our sample size.

Quantitative variables

Input and output data were aggregated for each DMU in both 2023 and 2024. MI, CU, and FS values were calculated for each DMU using DEA-Solver-Professional Version 12.1 (Saitech, Inc., Tokyo, Japan) [6,8]. These indices served as the primary outcome measures, reflecting productivity changes from 2023 to 2024.

All surgeons from the 10 included specialties were assigned MI, CU, and FS values. Natural logarithmic transformations of these indices were performed to enable interpretation in percentage terms [15-17]. A natural logarithm of MI greater than, equal to, or less than zero indicated increased, unchanged, or decreased productivity from 2023 to 2024, respectively. Similarly, positive logarithmic values of CU and FS reflected improvements in efficiency and technology. The natural logarithm of MI equals the sum of the logarithms of CU and FS [16,17].

The 10 surgical departments analyzed were as follows: cardiovascular surgery, emergency surgery, general surgery, neurosurgery, obstetrics and gynecology, orthopedics, otolaryngology, plastic surgery, thoracic surgery, and urology. For each specialty, the means and 95% confidence intervals of the logarithmic MI, CU, and FS values were calculated [17].

Statistical methods

Statistical analyses were conducted using Excel Statistics Software (Social Survey Research Information Co., Ltd., Tokyo, Japan). One-sample t-tests were used to determine whether the natural logarithms of MI, CU, and FS differed significantly from zero [18,19]. Statistical significance was defined as a p-value less than 0.05.

## Results

Participants

**Figure 1:**
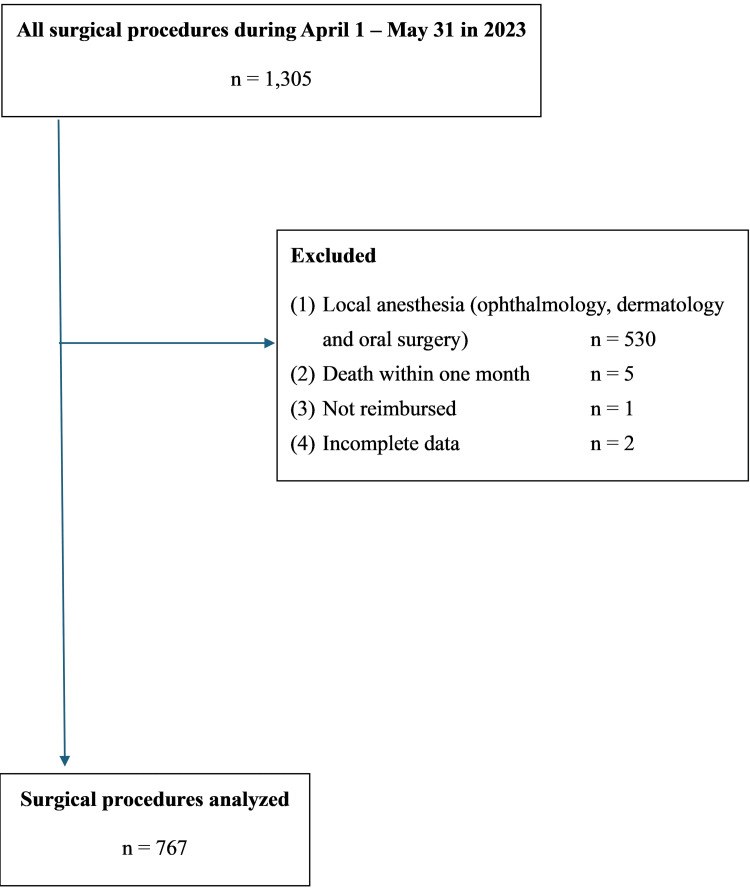
Flow diagram of surgical procedures in 2023 (before workstyle reform)

**Figure 2:**
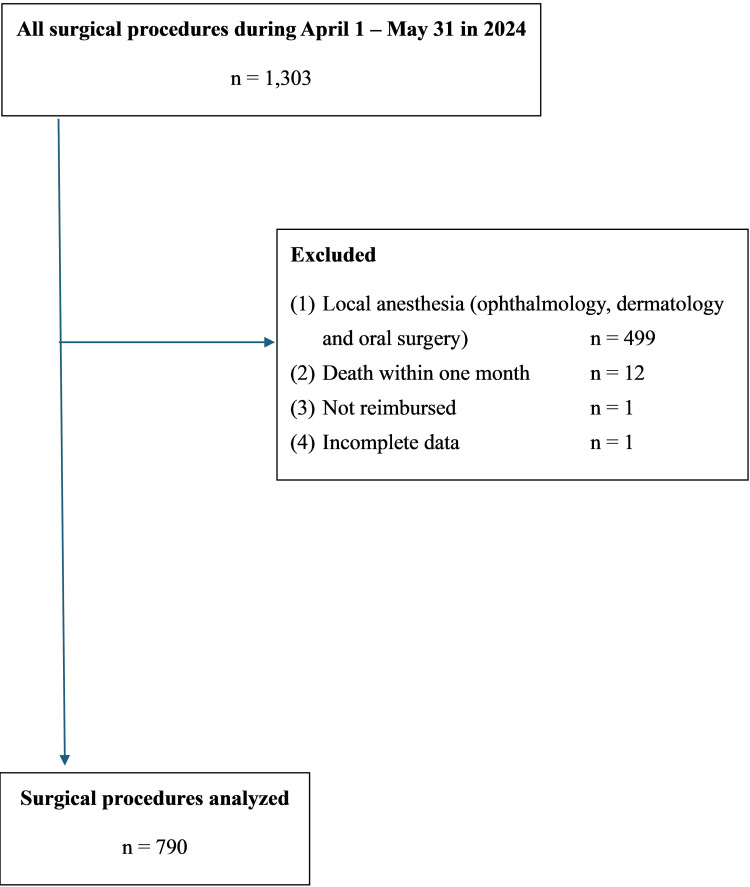
Flow diagram of surgical procedures in 2024 (after workstyle reform)

A total of 1,557 surgical procedures were included in the analysis, comprising 767 cases from 2023 and 790 from 2024. These surgeries were performed by 72 individual surgeons between April 1 and May 31 of each year (Figure 1 and Figure 2).

Descriptive data and outcome data

Surgical characteristics for the two study periods are presented in Table 1 and Table 2. In 2023, the average number of assisting doctors per procedure was 2.4, compared to 2.3 in 2024. The mean surgical time was 144 minutes in 2023 and increased slightly to 147 minutes in 2024. The average surgical fee per case was $2,748 in 2023, compared to $2,690 in 2024.

**Table 1:** Characteristics of surgery in 2023 (before workstyle reform) Assistants/case, surgical duration/case, and fee/case are expressed in means. The monetary values of surgical fees were converted to US dollars at $1 = 150 yen. DMU: decision-making unit

Specialty	DMUs	Cases	Assistants/case	Surgical duration/case (minutes)	Fee/case (US dollars)
Cardiovascular surgery	4	77	3.2	176	4,769
Emergency surgery	9	137	2.4	119	2,300
General surgery	15	125	2.4	159	2,635
Neurosurgery	7	34	2	215	5,564
Obstetrics and gynecology	9	95	1.9	119	1,926
Orthopedics	13	165	2.5	128	2,330
Otolaryngology	5	34	1.6	144	1,565
Plastic surgery	4	27	1.6	146	1,414
Thoracic surgery	3	26	2.5	198	4,336
Urology	3	47	2.9	155	2,748
All surgical procedures	72	767	2.4	144	2,748

**Table 2:** Characteristics of surgery in 2024 (after workstyle reform) Assistants/case, surgical duration/case, and fee/case are expressed in means. The monetary values of surgical fees were converted to US dollars at $1 = 150 yen.

Specialty	Cases	Assistants/case	Surgical duration/case (minutes)	Fee/case (US dollars)
Cardiovascular surgery	74	3.4	163	4,837
Emergency surgery	118	2.2	114	1,819
General surgery	125	2	198	2,890
Neurosurgery	38	2.2	250	7,087
Obstetrics and gynecology	94	2.4	128	1,930
Orthopedics	189	2.2	115	2,073
Otolaryngology	32	1.7	136	1,604
Plastic surgery	39	2	150	1,600
Thoracic surgery	23	2.5	160	4,221
Urology	58	3.1	144	2,380
All surgical procedures	790	2.3	147	2,690

Main results

The natural logarithms of the Malmquist Index (MI), catch-up (CU) effect, and frontier-shift (FS) effect are summarized in Table 3. The overall change in productivity across all surgical procedures was not statistically significant (p = 0.39), indicating no meaningful difference in productivity between 2023 and 2024. Subgroup analysis by specialty also revealed no statistically significant changes in productivity within any individual surgical department.

**Table 3:** Percent changes from 2023 (before workstyle reform) to 2024 (after workstyle reform) of productivity, catch-up effect, and frontier-shift effect of all surgeons and of each surgical specialty The values are expressed as mean (95% confidence interval). * indicates that the value is significantly different from 0 (p < 0.05).

Specialty	Productivity	Catch-up	Frontier-shift
Cardiovascular surgery	-8.1 (-29.7-13.5)	-22.8 (-42.5-3.0)	+14.7 (-1.3-28.0)
Emergency surgery	-23.2 (-51.6-5.2)	-28.2 (-56.0-0.4)	+5.0 (-3.2-13.2)
General surgery	-9.9 (-33.8-14.0)	-8.1 (-31.8-15.6)	-1.8 (-5.1-1.5)
Neurosurgery	+37.3 (-22.1-96.7)	+35.4 (-21.0-91.8)	+2.0 (-7.6-11.6)
Obstetrics and gynecology	-26.3 (-102.0-49.4)	-25.4 (-98.9-48.1)	-0.9 (-7.4-5.6)
Orthopedics	-24.2 (-66.7-18.3)	-19.2 (-64.8-26.5)	-5.0 (-9.7--0.3)*
Otolaryngology	+27.7 (-19.1-74.5)	+19.3 (-27.3-65.9)	+8.3 (-1.5-18.1)
Plastic surgery	+12.7 (-22.6-48.0)	+9.2 (-13.7-32.1)	+3.4 (-9.5-16.3)
Thoracic surgery	+20.1 (-19.1-59.3)	+17.8 (-17.4-53.1)	+2.3 (-7.3-11.9)
Urology	-12.0 (-30.6-6.6)	-13.6 (-52.8-25.6)	+1.7 (-20.8-24.2)
All surgeons	-6.5 (-21.2-8.2)	-7.6 (-22.3-7.1)	+1.2 (-1.15-3.6)

Other analyses

The CU effect, representing changes in efficiency, was not significantly different from zero across all procedures (p = 0.31), suggesting no efficiency gains or losses between the two years. Subgroup analysis showed that no surgical specialty experienced a statistically significant change in efficiency.

Similarly, the FS effect, indicating shifts in the technological frontier, showed no significant change across all procedures (p = 0.35), implying no overall technological advancement or regression between 2023 and 2024. However, subgroup analysis identified a significant negative FS effect in orthopedics (p = 0.049), suggesting a decline in relative technological progress in that specialty during the study period.

## Discussion

Key results

The findings presented above indicate that the hypothesis proposed in the Introduction was not confirmed, as the workstyle reform for physicians did not lead to a significant improvement in surgical total factor productivity during the study period. No statistically significant difference in surgical productivity was observed between the periods before and after the reform. Furthermore, neither the frontier-shift effect nor the catch-up effect showed a significant change. To our knowledge, this study is the first to examine the impact of physicians’ workstyle reform on changes in surgical productivity using real-world surgical data.

Interpretation

The causes for the findings above are impossible to identify from the present study because the MI model does not provide either clinical or managerial reasons for the numbers it calculated. Since the present study was a retrospective observational study, we can only speculate on some reasons. First, although overtime work was limited by the workstyle reform, the upper limit is known to pose significant risks to doctors’ health [1]. It is speculated that the workstyle reform did not improve doctors’ health and did not increase their surgical productivity. Second, the two-month study periods in each year might have been too short for the workstyle reform to take effect. The productivity changes from the workstyle reform might become clear if our study period was longer than two months each year. However, the productivity changes may be due to both workstyle reform and fee schedule revision [11,12]. It would be difficult to distinguish these two effects and to evaluate the pure effects of the workstyle reform. Further study will be necessary to evaluate the long-term effects of the workstyle reform for doctors. Third, on the contrary, there was no significant productivity change in 2024 because the hospital had prepared for the implementation of the workstyle reform before April 2024. It had started to collect data on doctors’ work hours before 2023 and discouraged all physicians from working too long. Fourth, the doctors who were most affected by the workstyle reform were junior doctors, who usually do not play any critical roles in surgery. They usually assist surgery following the senior surgeons’ advices. Even if junior doctors’ health improved and their risk of medical errors reduced, the workstyle reform would not lead to any improvement in surgical productivity that was measured in the present study. This might be a reason why surgical productivity did not change because it mostly depends on senior surgeons’ efforts.

There was only one surgical specialty that showed significant changes in FS effects during the study periods. Orthopedics significantly regressed in the FS effect from 2023 to 2024 (p = 0.049). The FS effect reflects technical changes that are not directly related to labor efficiency [8]. The technology in orthopedics declined on average by 5% from 2023 to 2024, whose clinical and managerial relevance is unknown. It is also impossible to specify any causes for this FS change from the present study.

Limitations

This study has several methodological limitations. First, we did not evaluate differences in surgical outcomes between 2023 and 2024. To maintain consistency in outcome quality, we excluded cases in which patients died within one month postoperatively. While this measure does not fully capture the quality of surgical outcomes, it was the only consistently available outcome metric across the diverse surgical procedures included in our analysis [14].

Second, although we attempted to minimize systematic bias by analyzing all eligible procedures during the same timeframe (April to May) across both years, the exclusion criteria may still have introduced some bias. Specifically, we omitted minor surgeries, procedures not covered under the surgical payment system, and cases with incomplete data. These exclusions may have narrowed the analytical scope and potentially influenced our findings.

Third, although we interpreted productivity changes using the Malmquist Index (MI) model through its decomposition into catch-up (CU) and frontier-shift (FS) effects, it is likely that other factors also contributed to the observed changes. The choice of inputs and outputs in the MI model, for instance, may have influenced the results. Hence, caution is warranted when attributing productivity changes to any single factor, as not all influences are captured within the MI framework.

Fourth, we did not make any case-mix adjustment between the two study periods. The analysis does not account for changes in the complexity or type of surgeries performed. Although Table 1 and Table 2 did not show any large differences in characteristics in surgery between 2023 and 2024, it is possible that a shift toward less complex (lower-fee) cases in 2024 may appear as a productivity decline in this model.

Generalizability

The generalizability of our findings may appear limited due to the study being conducted at a single large academic hospital in Tokyo. However, analyzing data from one institution offers certain advantages. Key resources such as operating room nursing staff and other ancillary services are standardized within a single facility, ensuring uniformity in operational conditions across all surgical departments. This internal consistency allows for fairer comparisons, as all departments are subject to the same institutional policies and resource constraints [20].

Teikyo University Hospital is categorized as “A Standard,” which has the strictest restriction on the overtime of the doctors and reflects the maximum effects of this workstyle reform [10]. A null result in the most strictly regulated setting does not necessarily predict the effect in less strict settings; those settings might see a different (positive or negative) impact. The effect was null under the strictest regulations, while the effects in other settings remain unknown.

## Conclusions

The workstyle reform in 2024 in Japan did not lead to a measurable change in fee-based surgical productivity in its first two months at this tertiary hospital. Neither frontier-shift nor catch-up effect was significant. Further study will be necessary to evaluate the long-term effects of the workstyle reform for doctors. Longer follow-up with richer data may yield different results.
